# Private collection: high correlation of sample collection and patient admission date in clinical microbiological testing complicates sharing of phylodynamic metadata

**DOI:** 10.1093/ve/vey005

**Published:** 2018-02-27

**Authors:** Ryan C Shean, Alexander L Greninger

**Affiliations:** 1 Department of Laboratory Medicine, University of Washington, 1616 Eastlake Avenue East, Suite 320, Seattle, WA 98102, USA; 2 Vaccine and Infectious Disease Division, Fred Hutchinson Cancer Research Center, 1100 Eastlake Avenue East, Seattle, WA 98102, USA

**Keywords:** collection date, admission date, Safe Harbor, privacy, protected health information, HIPAA

## Abstract

Infectious pathogens are known for their rapid evolutionary rates with new mutations arising over days to weeks. The ability to rapidly recover whole genome sequences and analyze the spread and evolution of pathogens using genetic information and pathogen collection dates has lead to interest in real-time tracking of infectious transmission and outbreaks. However, the level of temporal resolution afforded by these analyses may conflict with definitions of what constitutes protected health information (PHI) and privacy requirements for de-identification for publication and public sharing of research data and metadata. In the United States, dates and locations associated with patient care that provide greater resolution than year or the first three digits of the zip code are generally considered patient identifiers. Admission and discharge dates are specifically named as identifiers in Department of Health and Human Services guidance. To understand the degree to which one can impute admission dates from specimen collection dates, we examined sample collection dates and patient admission dates associated with more than 270,000 unique microbiological results from the University of Washington Laboratory Medicine Department between 2010 and 2017. Across all positive microbiological tests, the sample collection date exactly matched the patient admission date in 68.8% of tests. Collection dates and admission dates were identical from emergency department and outpatient testing 86.7% and 96.5% of the time, respectively, with >99% of tests collected within 1 day from the patient admission date. Samples from female patients were significantly more likely to be collected closer to admission date that those from male patients. We show that PHI-associated dates such as admission date can confidently be imputed from deposited collection date. We suggest that publicly depositing microbiological collection dates at greater resolution than the year may not meet routine Safe Harbor-based requirements for patient de-identification. We recommend the use of Expert Determination to determine PHI for a given study and/or direct patient consent if clinical laboratories or phylodynamic practitioners desire to make these data available.

## 1. Introduction

Rapid sequencing and phylodynamic tracking of viral and bacterial isolates have recently become a staple in tracking viral transmission and outbreaks. In the past 5 years, multiple collaborative efforts and wet-lab protocols have been described to obtain clinical materials and sequence whole genomes in hours to days (Carroll et al. 2015; Greninger et al. 2015b; Park et al. 2015; Quick et al. 2016; Kozyreva et al. 2017). These efforts range from international pandemics and smoldering state/providence-wide surveillance to local hospital-acquired or ward-based outbreaks (Gardy et al. 2011; Snitkin et al. 2012; Gire et al. 2014; Carroll et al. 2015; Roach et al. 2015; Greninger et al. 2015c; Kozyreva et al. 2016; Naccache et al. 2016; Thomson et al. 2016; Greninger et al. 2017b, c; Grubaugh et al. 2017). The use of genomic data in bacterial food-borne outbreaks has been associated with a faster determination of source and fewer persons associated with each outbreak (Jackson et al. 2016). Although the need for genomic data from infectious pathogens on a clinical level is limited, it is clear that we are at the beginning of a new age of infectious disease surveillance and response with great interest in ‘friction-free’ approaches to data sharing (Gardy et al. 2015). Whereas 5 years ago, there was little to no whole genome sequencing performed in public health laboratories, now more than 4,000 food-borne bacterial isolate genomes are uploaded each month to the Food and Drug Administration GenomeTrakr database by public health laboratories (Allard et al. 2016). Clinical labs and researchers can digitize stocks of virus at a cost of tens of dollars per isolate, unlocking novel pathogen biology at a local level (Greninger et al. 2015a, 2017a,d; Ogimi et al. 2017). Publicly deposited pathogen genomic data and metadata are forever available and searchable online, cumulatively adding to the world’s database of descriptive infectious diseaes epidemiology.

As the ease and rate of genomic data deposition has grown, pathogen metadata has become increasingly important. When depositing a viral or bacterial isolate sample for a clinical or host associated sample, the National Center for Biotechnology Information (NCBI) BioSample database requires organism name, strain/isolate name, collection location, collection date, isolation source, host disease, and latitude and longitude, but also allows for deposition of host age, host sex, host health state, host description, and antibiotic resistance metadata among other specimen identifiers (Federhen et al. 2014). Although privacy concerns for infectious pathogen genomic data and metadata are less than those associated with human genomic data, there are still considerable privacy issues at hand (Lippert et al. 2017). Naturally, pathogen metadata directly comes from patient metadata. The ability to impute human relations via pathogen genomic data and phylodynamics also raises serious privacy concerns as to what constitutes protected health information (PHI). Because pathogen sequence includes a positive clinical testing result by definition, pathogen metadata can be considered clinical metadata. The desire to build better phylodynamic models creates a demand for increasing amounts of pathogen metadata with higher resolution. Higher resolution of analysis corresponds to greater precision in identifying subjects. In addition, the number of laboratories sequencing isolates, depositing metadata, and participating in phylodynamic analyses is rapidly increasing and many laboratories may not be as aware of potential privacy concerns. Setting standards in this area is now especially critical.

In the United States the Deparment of Health and Human Services regulations name 18 individual identifiers that must be removed from datasets in order to be considered ‘de-indentified’ under the Safe Harbor method to be in accordance with the Privacy Rule of the Health Insurance Portability and Accountabiltiy Act (HIPAA) (Table 1). Alternatively, under the Expert Determination method, such identifiers may be included in released metadata if a qualified expert statistician deems them to be non-identifiable and the local Institutional Review Board (IRB) agrees. Finally, the IRB may grant permission for release of identifying metadata if direct patient consent is obtained to release such information. Public health entities have special provisions to communicate these identifiers in the context of a public health emergency (OCR 2008).

**Table 1.** List of 18 Protected Health Identifiers required to be removed to deidentify data under Safe Harbor method (reprinted from Office of Civil Rights Guidance on De-identification of PHI, 26 November 2012).

Protected health identifiers
Names
All geographical subdivisions smaller than a State, including street address, city, county, precinct, zip code, and their equivalent geocodes, except for the initial three digits of a zip code, if according to the current publicly available data from the Bureau of the Census: 1, The geographic unit formed by combining all zip codes with the same three initial digits contains more than 20,000 people; and 2, The initial three digits of a zip code for all such geographic units containing 20,000 or fewer people is changed to 000.
All elements of dates (except year) for dates directly related to an individual, including birth date, admission date, discharge date, date of death; and all ages over 89 and all elements of dates (including year) indicative of such age, except that such ages and elements may be aggregated into a single category of age 90 or older.
Phone numbers
Fax numbers
Electronic mail addresses
Social Security numbers
Medical record numbers
Health plan beneficiary numbers
Account numbers
Certificate/license numbers
Vehicle identifiers and serial numbers, including license plate numbers
Device identifiers and serial numbers
Web Universal Resource Locators (URLs)
Internet Protocol (IP) address numbers
Biometric identifiers, including finger, and voice prints
Full face photographic images and any comparable images
Any other unique identifying number, characteristic, or code (note this does not mean the unique code assigned by the investigator to code the data).

Sample collection date and location are not explicitly named in the 18 protected heath identifiers. However, dates associated with patient care such as admission date and discharge date at finer resolution than the year are considered patient identifiers. Locations at greater resolution than the first three zip code numbers or any subdivision smaller than a state are also considered protected information. For pathogens that evolve in days to weeks, providing only the year of collection based may lead to less precise predictive evolutionary models which lessen their public health impact. Alternatively, even with less detailed data deposition, finer resolution of the collection date may be imputed based on evolutionary models of genomic data and metadata available for all isolates of a given pathogen.

Here we examine the relationship between sample collection dates and admission dates. Under the Safe Harbor method of de-identification associated with the HIPAA Privacy Rule, admission date is considered PHI. If a specimen collection date is deposited in NCBI Genbank, to what degree has protected health information been released? Using more than 7 years of microbiological testing and over 270,000 unique results, we show that PHI in the patient’s hospital admission date can be confidently imputed from sample collection date. The correlation between these dates varies by different settings of care, with much higher levels of concordance associated with emergency department (ED) and outpatient care—where >99% of sample collections occurred within 1 day of admission. Greater effort must be made to anonymize, determine the identifiability of pathogen metadata, and/or obtain consent for the collection and publication of these data.

## 2. Methods

### 2.1 Clinical testing dataset

The University of Washington (UW) Laboratory Medicine Department is a large academic clinical laboratory medicine service that serves the University of Washington Medical Center (UWMC), Harborview Medical Center (HMC), the Seattle Cancer Care Alliance, Valley Medical Center, Northwest Hospital and Medical Center, and UW Neighborhood Clinics. The combined enterprise covers ∼63,000 inpatient admissions, 200,000 emergency room visits, and 1.6 million outpatient visits each year (*UW Medicine Board Annual Financial Report, FY 2016* n.d.).

Because we were chiefly interested in the use of patient identifiers in the context of phylodynamic analyses, we attempted to restrict our analysis to clinical microbiology testing for which a positive result was obtained, as only positives could be sequenced. We obtained all bacterial and fungal culture test orders for which a positive result/isolate was recovered as well as all viral testing that was ordered and sent to UWMC Microbiology laboratory, HMC Microbiology laboratory, and UW Virology between 1 January 2010 and 2017 (Supplementary Table S1). Of note, all virology test orders—positive or negative—were analyzed because coding of the virology results could not be readily restricted to positives based on reporting of virology results.

The original identifiers obtained included name, medical record number, date of birth, sex, location of patient when the sample was collected, the classification of patient encounter (inpatient, outpatient, emergency room, or outside), sample collection date, patient admission date, patient discharge date, accession number, order code, order name, test code, test name, test result, and result time. All identifiers except sex, location, classification, admit date, discharge date, and collection date were expunged from the working dataset, although the original dataset was retained for chart review of select cases to ensure data validity.

Cleaning of data was first performed by allowing only one entry for each laboratory information system sample accession number, which collapsed multiple clinical tests from a single sample into one test but did not collapse data by patient. For instance, if a patient had one sample taken on Day 1 for two viral tests and then another sample was collected the next day for three bacterial tests this would translate into two data points where the sample was taken on Day 1 and one data point on Day 2. The data were then quality-controlled by removing all entries that had any empty or nonsensical values for any of the kept metadata (e.g. alphanumeric strings for admission date or unknown patient sex). Chart review of these entries revealed that they were the result of clinical staff competency testing. These entries were subsequently removed from further analysis. We then removed all testing for which the collection date occurred after the discharge date or before the admission date, as all sample collections should be made between an admission date and discharge date for a given clinical encounter. The above quality control steps were decided upon after chart review of select outliers revealed that the above criteria were also highly likely to be associated with reference lab testing; therefore, we also removed all reference lab testing due to inability to confidently assign admission dates to samples sent from outside hospitals. We also quality-controlled the data by examining outliers such as the longest patient stays for ED and outpatient settings. The long patient stay lengths were all valid, and the ED stays were in all cases boarding patients in the ED due to bed unavailability based on chart review.

### 2.2 Statistical analyses

Because collection times were reported to the minute but admission dates were only reported to the day, we restricted analysis to the number of days between collection date and admission date, with zero indicating identical admission and collection dates. In order to show the degree to which collection dates and admission dates were related on a continuous basis, cumulative distribution curves were calculated based on the number of days separating the collection date and admission date for each sample collected. Distributions were plotted using the ggplot2 package for R (Wickham 2009). Two-sample Kolmogorov-Smirnov (KS) tests were used to compare cumultative distributions and two-sample proportion tests were used to compare specific cumulative proportions at a point in time.

## 3. Results

Information from a total of 286,150 samples was analyzed from microbiological testing at the UW Laboratory Medicine Department from 2010 to 2017. Of these, 14,381 samples (5.0%) were removed due to staff testing, computer records testing, and conflicting admission/discharge/collection dates as described above in the Methods. A total of 271,769 samples were included for downstream analysis of collection date and admission date. ED samples accounted for 32,161 (11.8%) of all samples, inpatient for 95,188 (35.0%) and outpatient for 144,420 (53.1%). Samples collected from female patients accounted for 138,970 (51.1%) samples, and male patient samples made up the remaining 132,799 (48.9%). Positive bacterial/fungal testing made up 74,857 (27.5%) of the samples analyzed and all viral testing comprised 196,912 samples (72.5%) (Table 2).

**Table 2.** Demographic characteristics of samples tested.

	Bacterial–fungal	Viral
Emergency Room	2,197	29,964
Inpatient	16,157	79,031
Outpatient	56,503	87,917

Bacterial–fungal	Female	Male

Emergency Room	967	1,230
Inpatient	6,976	9,181
Outpatient	24,883	31,620

Viral	Female	Male

Emergency Room	15,516	14,448
Inpatient	33,391	45,640
Outpatient	57,237	30,680

**Figure 1.**
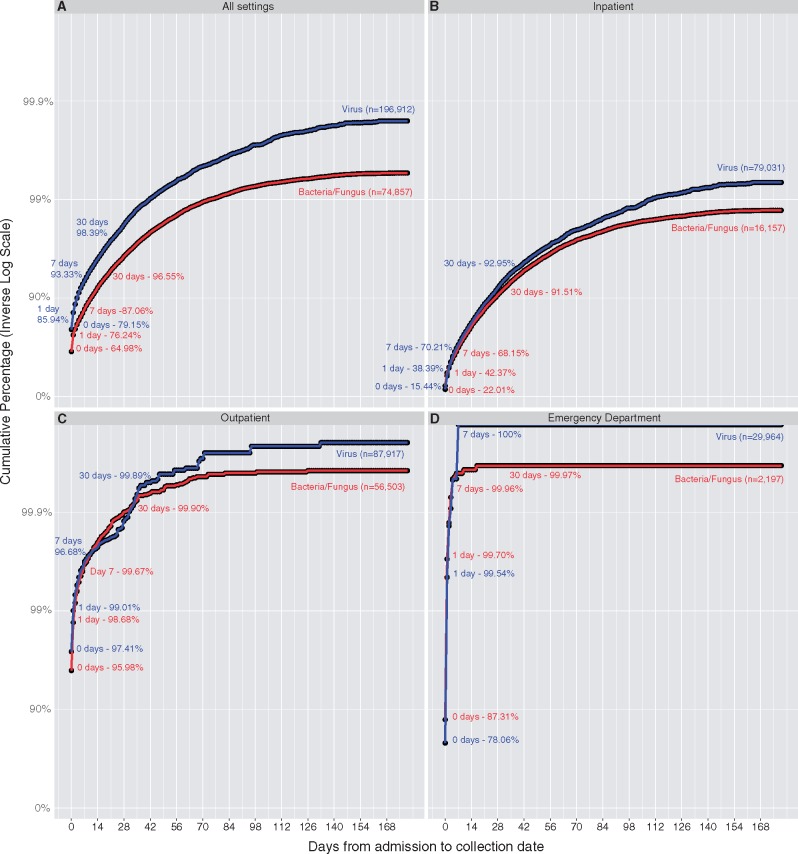
High concordance of collection date and admission date for positive microbiological testing in multiple care settings. Cumulative probability curves of the collection date being with X days of the admission date are depicted for all settings (A), inpatient (B), outpatient (C), and emergency department (D). All positive microbiological tests (bacteria/fungus) and all virology tests (virus) that had collection dates between 2010 and 2017 from the University of Washington Laboratory Medicine Department are shown. Overall, positive microbiological tests had a collection date that was within 1 day of a patient’s admission date 78.8% of the time. Viruses were significantly more likely to be collected closer to the admission date across all settings (*P* = 2.2e-16). Cumulative percentages for days 0, 1, 7, and 30 are depicted to highlight ability to impute admission dates exactly, within 1 day, 1 week, and 1 month based on the collection date. Cumulative percentages are plotted on a logarithmic scale due to the very high likelihood of collection date being associated admission date for outpatient and emergency department testing (>98.5% of collection dates were within 1 day of admission date for each location). Two-sample KS testing was performed on all cumulative distributions. Bacteria compared with viruses was significantly different across all locations (*P* < 2.2e-16). Comparing viruses across all locations and all bacteria across all locations also revealed that differences between the distributions were significant (*P* < 2.2e-16).

Across all samples for which data were kept, 68.8% of samples had a collection date that was identical to the patient’s admission date (Fig. 1A ). Furthermore, 78.8% of samples were collected within 1 day of the admission date. Of all samples analyzed, only 20 samples (0.01%) were collected more than 1 year from the patient’s admission date, 279 (0.1%) more than 180 days from the admission date, and 26,542 (9.8%) more than 60 days from the admission date. For 99.9% of samples the collection date was within half a year of the admission date, far greater than the year resolution defined under Safe Harbor method of de-identification. Strikingly, more than 97% of samples were collected within 1 month of the patient admission date.

In order to understand the degree to which additional pieces of sample metadata can affect the ability to more confidently impute patient admission date from sample collection date, we performed additional subgroup analyses based on availability of sample metadata. Viral testing collection dates were more strongly correlated with admission dates than for positive bacterial or fungal culture samples (*P*-value < 2.2e-16). Across all locations, collection dates were identical to patient admission dates for 79.2% of viral tests, while they were identical for 65.0% of positive bacterial and fungal cultures.

Virology test coding did not permit ready identification of only positive test results. To check the impact of examining all virus testing versus only viral positives, we extracted 16,434 unique samples for which the test results field either contained ‘positive’ or a quantity. In this subset, 14,133 (86.0%) of samples were collected on the same day as the admission date and 14,994 (91.2%) of the samples were collected within 1 day of admission. The distribution of this subset of viral positives was significantly different compared with all virus testing (*D* = 0.373, *P* = 4.86e-11). A likely explanation for this phenomena is the increased prevalence of positive samples being collected in the outpatient setting (83.5%) as compared with in the larger dataset (53.1%). These discrepancies can likely be explained by continued monitoring and sampling of patients with chronic viral infections. For example, outpatient clinics may perform routine viral load monitoring in the setting of chronic viral infection. Thus, by examining all viral tests and not just positives we are likely biased toward larger discrepancies between collection date and admission date.

We next separated bacterial/fungal and virology testing by the type of patient encounter: inpatient, outpatient, and ED (Fig. 1B–D). Unsurprisingly, almost all of the differences between collection date and admission date were due to inpatient testing. Across all inpatient testing, the patient admission date was identical to sample collection date for 22.0% of bacterial samples and 15.4% of viral testing samples. However, sample collection dates were within 30 days of inpatient admission dates for 91.5% of bacterial/fungal samples and 93.0% of viral samples. Presumably, most of the microbiological testing for inpatients in which admission date and collection date matched was ordered from the ED. Across both bacterial and viral samples, 99.7% of outpatient samples and 99.5% of ED samples were collected within 1 day of admission date. For outpatient testing, sample collection dates and patient admission dates were identical for 96.0% of positive bacterial tests and 97.4% of virology tests (Fig. 1C).

**Figure 2.**
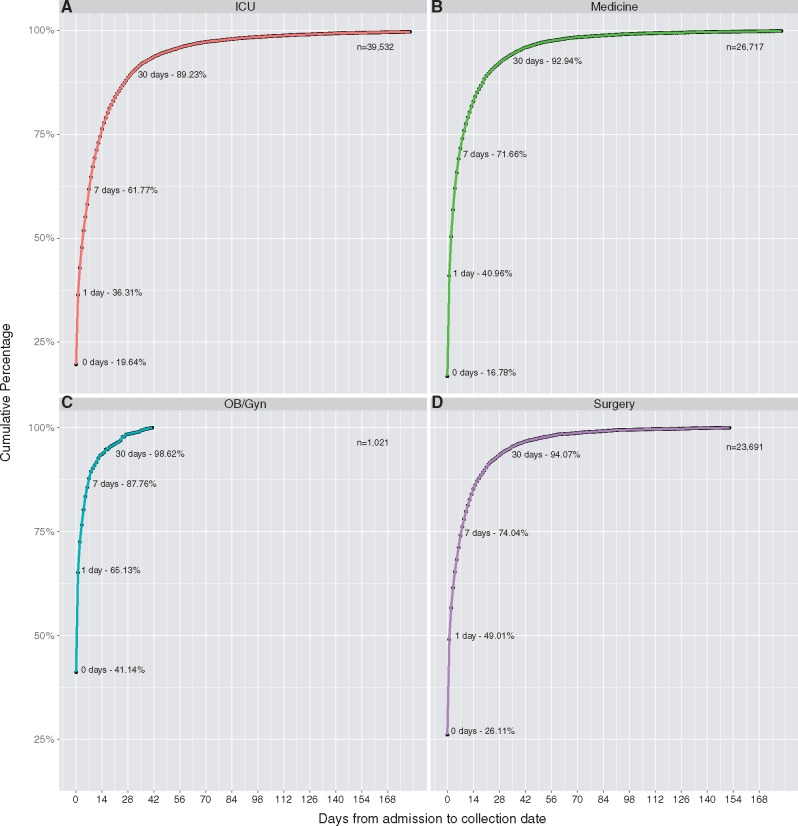
High concordance of collection date and admission date in OB-Gyn testing. Cumulative probability curves of the collection date being within X days of the admission date are shown for inpatients grouped into the following four categories—ICU (A), Medicine (B), OB/Gyn (C), Surgery (D). Bacterial/fungal culture and viral tests are combined for this figure. ICU had the lowest percent of admission dates being within 1 day of collection date at 36.31%. OB/Gyn had the largest percent of collection dates dmission dates being within 1 day of collection at 65.13%. Cumulative percentages for Days 0, 1, 7, and 30 are shown to highlight ability to impute admission dates based on collection dates with that many days of accuracy. The cumulative distribution of days separating collection date and admission date differed significantly between OB/Gyn and each of the other services: ICU (*P* = 0.0022), Medicine (*P* = 3.62e-05), and Surgery (*P* = 0.0027). ICU, Medicine, and Surgery were not found to have significantly different cumultative distributions between each other (*P* > 0.2).

Because the majority of discrepancies in collection and admission dates occurred for inpatient testing, we further subdivided inpatient results based on the type of ward from which the sample was collected: intensive care unit (ICU); medicine and medical subspecialities; obstetrics/gynecology (OB/Gyn); or surgery and surgical subspecialities (Fig. 2A–D). We were able to classify 90,961 (95.6%) of all 95,188 inpatient samples. Obstetrics and gynecology had the highest percentage of samples collected within 1 day of the admission date at 65.1%, next highest was surgery at 49.0%, followed by medicine at 41.0%, and ICU at 36.3%. Pairwise two-sample KS tests were performed for all combinations of locations. The only significant difference in distributions was between OB/Gyn and all other categories—ICU (*D* = 0.31, *P* = 0.002), medicine (*D* = 0.039, *P* = 3.619e-05), and surgery (*D* = 0.317, *P* = 0.002). All other pairwise comparisons of distributions resulted in *P*-values > 0.002.

**Figure 3.**
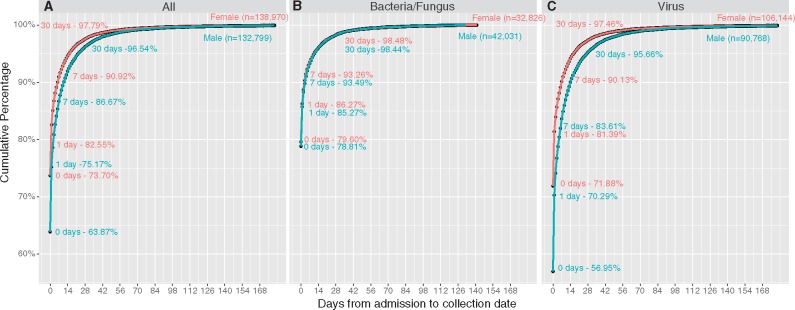
Higher concordance of collection date and admission date for females than for males. Cumulative probability curves of the collection date being within X days of the admission date are shown for all locations and sexes grouped by the test type – all tests combined (A), bacterial/fungal culture positives (B), all viral testing (C). Samples from women were significantly more likely to be collected on the exact admission date (73.7 versus 63.9%, *P* = 2.2e-16) and closer to the admission date (*D* = 0.089916, *P* = 0.0137) than those from men.

We next examined differences between collection and admission dates by patient gender and across time (Fig. 3). Samples from women were significantly more likely to be collected on the exact admission date (73.7% versus 63.9%, *P* = 2.2e-16) and closer to the admission date (*D* = 0.089916, *P* = 0.0137) than those from men. All-by-all pairwise two-sample KS testing for cumulative distribution by year was also performed and Bonferroni corrected for 15 total tests. The lowest Bonferroni-corrected *P*-value obtained was 0.105 indicating that distributions were not significantly different year-by-year.

## 4. Discussion

In this study, we show that sample collection dates associated with microbiological testing at a large tertiary hospital are highly correlated with patient admission date, protected health information specifically named by the HIPAA Privacy Rule. With the addition of one piece of metadata such as outpatient or ED testing, the admission date could be imputed to within 1 day for over 99% of samples with a listed collection date. Even for inpatient testing, collection dates allowed imputation of the admission date to the resolution of a week for >70% of samples. Indeed, the average length of stay is ∼1 week in most of the hospitals that send clinical specimens to our clinical laboratory (*UW Medicine Board Annual Financial Report, FY 2016* n.d.). We also note that because discharge date, also PHI date specifically named in HIPAA Privacy Rule, was not included in the analysis, the above findings represent the lower bound of what might be imputed from sample collection dates.

Metadata such as the setting of care can potentially be gleaned from the type of microbiological organism sequenced. Pathogens of high public health importance—for which much high impact phylodynamic analysis has recently been performed—are likely to be tested for early in admission and may show higher correlations with admission date. For instance, both Ebola virus tests sent to our clinical laboratory in 2014 were sent the day of admission.

Whole genome sequencing is increasingly performed in the clinical microbiology laboratory. Sequencing of clinical microbiological samples has been used to rule-in or rule-out infectious diseases through metagenomics, to detect antimicrobial or antiviral resistance, and to impute the transmission source (e.g. to rule-in or rule-out hospital-acquired outbreaks) (Capobianchi, Giombini, Rozera 2013; Greninger et al. 2015a, 2017b,c; Grubaugh et al. 2017; Naccache et al. 2014, 2016; Simon-Loriere et al. 2015). In this way, sequence data that is obtained for detection of antimicrobial resistance to inform direct patient care can also be used for public health measures and infection prevention. Based on the continuing logarithmic growth of data available in Genbank and other NCBI databases, we foresee a future of rich infectious disaese genomic databases that allow imputation of local and global transmission relationships of pathogens, limited chiefly by the evolutionary rate of the organism in question. The emergence of these data creates a pressure for clinical laboratories to share sample metadata, and a demand for community guidance as to the manner in which such clinical metadata can appropriately be shared. Indeed, the question posed here originated from a local decision by our clinical laboratory and IRB to follow the Safe Harbor method of de-identification for samples sequenced in the clinical laboratory and to deposit only year data for sample collection dates (Ogimi et al. 2017).

Practioners can reasonably doubt that a bacterial or viral test result, a genomic sequence, and an associated date constitute PHI. Such data may best be described as a quasi-identifier (Emam, Rodgers, Malin 2015). However, in the United States, the Safe Harbor provision of the HIPAA privacy rule requires the de-identification of high-resolution dates from patient information to anonymize such data. Limited datasets that include some date and location information can be disclosed to non-covered entity recipients with an explicit data use agreement (Pace, Staton, Holcomb 2005). However, publicly available databases such as NCBI Genbank do not meet this standard. Phylodynamic models can be constructed based on full metadata provided to the investigators, and then outbreak dates can be adjusted relative to the start of the outbreak (Greninger et al. 2017b,c). Although this effectively deals with patient privacy issues, it does not create generalizable models of pathogen evolution nor is it necessarily reproducible by other researchers who do not have access to the full dataset.

Since these regulations allow for Expert Determination of whether specific information can be considered identifiable, we encourage clinical laboratories and phylodynamics practioners to take advantage of this avenue for evaluating their studies if they want to upload exact collection date associated with patient care (Sarpatwari et al. 2014). Explicit patient consent to share such data may also be obtained, although blanket consent forms often include provisions that patient data will be anonymized before release (Greninger et al. 2017d). Alternatively, the clinical, scientific, and public health communities could determine that the benefits outweight the costs and change regulations around high-resolution sample collection date sharing (Wartenberg and Thompson 2010).

The degree of identifiability of a given patient or case can be fundamentally unknowable, especially when there exists a continuum of other metadata sources either on the Internet or in the community (Sweeney 2013). Genomic sequences in NCBI’s Genbank also frequently feature other sample collection information, like sex, age, and sampling hospital which can be combined with other information inherent in the sequence to reduce the statistical obscurity of these data by a very large degree. Different organisms, from Ebola virus and *Pandoraea apista* to human herpesvirus 2, may have different degrees of identifiability and different degrees of stigma associated with them. All of these give a strong rationale for the growing role and importance of Expert Determination for de-identification of data (Meyers et al. 2017).

Our study is limited in that our simple model effectively only uses one or two variables to impute admission date. We sought only to show that PHI was readily imputable from sample collection dates. These results do not indicate whether these patients are in fact identifiable nor how much metadata is required to make them identifiable. Indeed, we did not attempt to use this information to identify actual individuals in this study. Nor have there been many studies on the re-identifiability from admission date, as there have been on date of birth (Golle 2006; Benitez and Malin 2010; Sweeney 2013). The answer to these questions often changes over time as increasing amounts of identifiable metadata become available (Liu, Musen, and Chou 2015). We also did not take into account reference laboratory testing due to inability to confidently ascertain admission dates from outside hospitals. As many clinical laboratories do not run large reference operations, it was unclear what generalizibility these data would offer to other laboratories. Our vantage point is also biased by a focus on the United States clinical testing and HIPAA Privacy Rule. Most recent phylodynamic analysis has been performed outside the United States where rules associated with patient data privacy may be interepreted differently (Faria et al. 2016). Indeed, assuming a predictable evolutionary rate for a given pathogen, one may be able to use phylodynamic modeling accurately impute the collection dates from samples where data was obscured to comply with local privacy and IRB requirements (Dudas et al. 2017).

In an era of big data, clinical laboratories are rich sources of clinical data and metadata, and we do not expect these questions to go away. Metadata deposition into large centralized databases for research purposes is one of a host of ethical issues associated with communicating patient data and metadata. Although academic journals increasingly require direct patient consent to publish case reports, pathologists are encouraged to post interesting histopathology, gross tissue, or laboratory medicine (e.g. Gram stain, blood smear) images on social media to enhance their own personal brand as well as that of a profession that is often found in the hospital basement (Crane and Gardner 2016; Brissette et al. 2017; Isom, Walsh, and Gardner 2017). Phylodynamic metadata is but one among many ethical issues and questions facing the clinical laboratory today.

## Ethics statement

This study was approved by the UW IRB (Study No. 00002488).

## Supplementary Material

Supplementary Data

## Data Availability

The line-item datasets generated and/or analyzed during the current study are not publicly available due to PHI but may be available from the corresponding author on reasonable request.
