# Supplementary material for: Private collection: high correlation of sample collection and patient admission date in clinical microbiological testing complicates sharing of phylodynamic metadata
Source: Virus Evol. 2018 Feb 27;4(1):vey005. doi: 10.1093/ve/vey005 (PMC5829646; doi:10.1093/ve/vey005)
Supplement: Supplementary Data [file vey005_supp.docx]

**Supplementary Table 1. Test codes analyzed in this study**

| Test Name | Grouping for this Study |
| --- | --- |
| R/O Acinetobacter Bacterial Culture only without Gram | Bacterial/Fungal |
| R/O Actinomyces Bacterial Culture and Sensitivity | Bacterial/Fungal |
| Adenoviruses by PCR Qualitative, Tissue or Cells | Viral |
| AFB Culture, Blood | Bacterial/Fungal |
| AFB Culture, Bone Marrow | Bacterial/Fungal |
| AFB Culture without stain | Bacterial/Fungal |
| AFB Culture with Stain | Bacterial/Fungal |
| Aspergillus fumigatus PCR | Bacterial/Fungal |
| Aspergillus fumigatus PCR, Tissue | Bacterial/Fungal |
| Parvovirus B19 by PCR Qualitative Tissue or Cells | Viral |
| Bacterial DNA detection from direct specimens and identification by amplification | Bacterial/Fungal |
| Bacteria (Non-AFB) Identification by Sequencing | Bacterial/Fungal |
| BK Virus by PCR Qualitative | Viral |
| BK Virus by PCR Quantitative | Viral |
| Blood Culture & Sensitivity, Isolator | Bacterial/Fungal |
| Blood Culture and Sensitivity | Bacterial/Fungal |
| Fungal Culture, Blood | Bacterial/Fungal |
| Isolator Blood Fungal Culture | Bacterial/Fungal |
| Blood Culture and Sensitivity (Line Draw) | Bacterial/Fungal |
| Blood Culture & Sensitivity Quantitative (Line | Bacterial/Fungal |
| Blood Culture & Sensitivity Quantitative | Bacterial/Fungal |
| Bone Marrow Culture and Sensitivity with Gram Stain | Bacterial/Fungal |
| Bone Marrow Fungal Culture with Direct Exam | Bacterial/Fungal |
| R/O Beta Strep Culture | Bacterial/Fungal |
| R/O Group B Beta Strep, Genital Culture | Bacterial/Fungal |
| CMV Qualitative by PCR, Tissue/Cells | Viral |
| Coccidioides species DNA detection from direct specimens and identification by amplification | Bacterial/Fungal |
| Cryptococcus neoformans and Cryptococcus gattii DNA detection from direct specimens and identificati | Bacterial/Fungal |
| CSF Bacterial Culture and Sensitivity (Special Anaerobic) with Gram Stain | Bacterial/Fungal |
| CSF Culture with Gram Stain | Bacterial/Fungal |
| CSF Fungal Culture | Bacterial/Fungal |
| Ear Culture & Sensitivity with Gram Stain | Bacterial/Fungal |
| EBV by PCR Qualitative, Tissue /Cells | Viral |
| Environ. C/S (General) | Bacterial/Fungal |
| Enterovirus/Parechovirus PCR | Viral |
| Eye Culture and Sensitivity with Gram | Bacterial/Fungal |
| Eye Fungal Culture with Direct Exam | Bacterial/Fungal |
| Herpes Simplex & Varicella Zoster FA | Viral |
| Fluid Bacterial Culture and Sensitivity (Special Anaerobic) with Gram Stain | Bacterial/Fungal |
| Fluid culture and Sensitivity with Gram (Orthopedic surgery specimen) | Bacterial/Fungal |
| Fluid Bacterial Culture in Blood Culture Bottles without Gram Stain | Bacterial/Fungal |
| Fluid Bacterial Culture and Sensitivity with Gram Stain | Bacterial/Fungal |
| Fluid Culture with Direct Fungal Exam | Bacterial/Fungal |
| Fluid Bacterial C/S in Blood Culture Bottles with Gram Stain | Bacterial/Fungal |
| Fungal DNA detection from direct specimens and identification by amplification | Bacterial/Fungal |
| R/O Gonorrhoeae without Gram | Bacterial/Fungal |
| Genital Bacterial Culture and Sensitivity (Routine) without Gram Stain | Bacterial/Fungal |
| Mycoplasma, Genital by PCR | Bacterial/Fungal |
| Genital Bacterial Culture and Sensitivity (Anaerobic) with Gram Stain | Bacterial/Fungal |
| Hepatitis B PCR Quantitative | Viral |
| HHV6 Quantitative by PCR | Viral |
| Human Herpes Virus Type 8 by PCR Qualitative Tissue or Cells | Viral |
| HHV8 PCR, Quantitative | Viral |
| HSV Qualitative by PCR, Tissue /Cells | Viral |
| HSV Quantitative by PCR | Viral |
| Herpes Simplex Virus Quantitative by PCR Reflex I, II Typing | Viral |
| JC Virus by PCR Quantitative | Viral |
| R/O Legionella Bacterial Culture & Sensitivity | Bacterial/Fungal |
| Lower Respiratory Bacterial Culture - R/O Anaerobes with GRAM | Bacterial/Fungal |
| Lower Respiratory Bacterial Culture and Sensitivity with Gram Stain | Bacterial/Fungal |
| Lower Respiratory Culture for Cystic Fibrosis | Bacterial/Fungal |
| Lower Respiratory Fungal Culture with KOH Exam | Bacterial/Fungal |
| Lower Respiratory Bacterial Culture, Quantitative with Gram Stain | Bacterial/Fungal |
| M avium complex PCR | Bacterial/Fungal |
| Minimum Inhibitory Concentration, Bacteria | Bacterial/Fungal |
| Mycoplasma pneumoniae by PCR | Bacterial/Fungal |
| R/O MRSA Bacterial Culture only without Gram | Bacterial/Fungal |
| Mycoplasma, Miscellaneous by PCR | Bacterial/Fungal |
| R/O Nocardia Culture with Modified AFB Smear | Bacterial/Fungal |
| Non Tuberculous Mycobacteria DNA detection from direct specimens and identification by amplification | Bacterial/Fungal |
| Pneumocystis DNA detection from direct specimens and identification by amplification | Bacterial/Fungal |
| Extended Respiratory Virus PCR Panel | Viral |
| Cytomegalovirus Rapid Detection | Viral |
| Respiratory Sinus C/S w/Gram | Bacterial/Fungal |
| Quantitative Respiratory virus follow-up by PCR | Viral |
| Skin Culture and Sensitivity with Gram Stain | Bacterial/Fungal |
| Skin, Hair, Nail or Vesicle Fungal Culture with KOH Exam | Bacterial/Fungal |
| R/O Staph aureus Bacterial Culture only without Gram | Bacterial/Fungal |
| Stool Bacterial Culture & Sensitivity for Enteric Pathogens | Bacterial/Fungal |
| MTB complex PCR | Bacterial/Fungal |
|  |  |
| Quantitative Intravascular Catheter Line or Tip Bacterial Culture Quantitative | Bacterial/Fungal |
| Tissue Bacterial Culture and Sensitivity with Gram | Bacterial/Fungal |
| Fungal Tissue Culture with KOH Direct Exam | Bacterial/Fungal |
| Tissue Culture & Sensitivity (special anaerobic, Ortho surg. specimen) with Gram | Bacterial/Fungal |
| Tissue Bacterial Culture & Sensitivity, Quantitative | Bacterial/Fungal |
| Toxoplasma gondii DNA detection from direct specimens and identification by amplification | Bacterial/Fungal |
| BK Virus by PCR Quantitative, Urine | Viral |
| Urine Culture and Sensitivity with Gram (Anaerobic) | Bacterial/Fungal |
| Urine Culture and Sensitivity with Gram | Bacterial/Fungal |
| Urine Fungal Culture without Direct Exam | Bacterial/Fungal |
| Urine Culture and Sensitivity without Gram | Bacterial/Fungal |
| Upper Respiratory Culture and Sensitivity without Direct Exam | Bacterial/Fungal |
| Upper Respiratory Fungal Culture with Direct Exam | Bacterial/Fungal |
|  | Viral |
| Herpes Group Culture and CMV Rapid Detection | Viral |
| Herpes Group Culture (HSV 1&2, CMV, VZ) | Viral |
| Herpes Group Culture and FA |  |
| Viral Culture Screen | Viral |
| Viral Culture and Rapid CMV Detection | Viral |
| R/O Vancomycin Resistant Enterococcus Bacterial Culture and Sensitivity | Bacterial/Fungal |
| Varicella Zoster Qualitative by PCR, Tissue or Cells | Viral |
| Varicella Zoster Quantitative by PCR | Viral |
| Wound Bacterial Culture and Sensitivity (Special Anaerobic) with Gram Stain | Bacterial/Fungal |
| Wound Culture (special anaerobic for Orthopedic surgery specimen) with Gram | Bacterial/Fungal |
| Wound Bacterial Culture and Sensitivity with Gram Stain | Bacterial/Fungal |
| Wound Fungal Culture with KOH Exam | Bacterial/Fungal |
| West Nile Virus by PCR Quantitative | Viral |
| R/O Yeast Culture with Direct Exam | Bacterial/Fungal |
| Yeast (Fungi) MIC Susceptibility Panel | Bacterial/Fungal |
| Zygomycete species DNA detection from direct specimens and identification by amplification | Bacterial/Fungal |
| Zika Virus by PCR | Viral |
| R/O Acinetobacter Bacterial Culture only without Gram | Bacterial/Fungal |
